# Engineered Fully Human Single-Chain Monoclonal Antibodies to PIM2 Kinase

**DOI:** 10.3390/molecules26216436

**Published:** 2021-10-25

**Authors:** Kanasap Kaewchim, Kittirat Glab-ampai, Kodchakorn Mahasongkram, Monrat Chulanetra, Watee Seesuay, Wanpen Chaicumpa, Nitat Sookrung

**Affiliations:** 1 Graduate Program in Immunology, Department of Immunology, Faculty of Medicine Siriraj Hospital, Mahidol University, Bangkok 10700, Thailand; kanasap.kaw@student.mahidol.edu; 2 Center of Research Excellence on Therapeutic Proteins and Antibody Engineering, Department of Parasitology, Faculty of Medicine Siriraj Hospital, Mahidol University, Bangkok 10700, Thailand; kittirat.gla@mahidol.edu (K.G.-a.); k.mahasongkram@gmail.com (K.M.); monrat.chl@mahidol.edu (M.C.); watee.see@gmail.com (W.S.); wanpen.cha@mahidol.ac.th (W.C.); 3 Biomedical Research Incubator Unit, Department of Research, Faculty of Medicine Siriraj Hospital, Mahidol University, Bangkok 10700, Thailand

**Keywords:** human scFv, phage display, PIM2 kinase, ATP-binding pocket, homology modeling, intermolecular docking

## Abstract

Proviral integration site of Moloney virus-2 (PIM2) is overexpressed in multiple human cancer cells and high level is related to poor prognosis; thus, PIM2 kinase is a rational target of anti-cancer therapeutics. Several chemical inhibitors targeting PIMs/PIM2 or their downstream signaling molecules have been developed for treatment of different cancers. However, their off-target toxicity is common in clinical trials, so they could not be advanced to official approval for clinical application. Here, we produced human single-chain antibody fragments (HuscFvs) to PIM2 by using phage display library, which was constructed in a way that a portion of phages in the library carried HuscFvs against human own proteins on their surface with the respective antibody genes in the phage genome. Bacterial derived-recombinant PIM2 (rPIM2) was used as an antigenic bait to fish out the rPIM2-bound phages from the library. Three *E. coli* clones transfected with the HuscFv genes derived from the rPIM2-bound phages expressed HuscFvs that bound also to native PIM2 from cancer cells. The HuscFvs presumptively interact with the PIM2 at the ATP binding pocket and kinase active loop. They were as effective as small chemical drug inhibitor (AZD1208, which is an ATP competitive inhibitor of all PIM isoforms for *ex vivo* use) in inhibiting PIM kinase activity. The HuscFvs should be engineered into a cell-penetrating format and tested further towards clinical application as a novel and safe pan-anti-cancer therapeutics.

## 1. Introduction

The proviral integration site of Moloney murine leukemia virus proteins (acronym PIMs) are kinases of the serine/threonine kinase family. PIMs composed of three different isoforms, i.e., PIM1, PIM2 and PIM3 [1,2]. The PIM2 encoded by *pim2* is involved in cell growth, survival and proliferation [3]. In human cells, a single *pim2* transcript gives rise to three PIM2 variants of molecular masses 34, 37 and 40 kDa due to in-frame alternative translation initiation sites; the three variants share an identical catalytic/kinase domain (residues 32–286) but differ at their N-termini [4]. The intracellularly expressed PIM2 is constitutively active regardless of cytokines or mitogenic signals [5]. PIM2 is known as a transcriptionally regulated apoptotic inhibitor [5] that functions independently of AKT, PI3K, mTOR signal transduction pathways [6]. PIM2 mediates survival signaling through phosphorylation of several pro-apoptotic proteins resulting in arrest of cell death. PIM2 phosphorylates BAD (Bcl-2 associated agonist of cell death) and reverses the pro-apoptotic property of BAD, hence preventing cell death [7]. PIM2 phosphorylates c-Myc to increase c-Myc stability and transcriptional activity [8]. PIM2 phosphorylates 4E-binding protein 1 (4E-BP1) which results in inhibition of the 4E-BP1 binding to eukaryotic translation initiation factor (eIF4E), leading to cap-dependent translation and inhibition of pro-apoptotic activity [5,9,10]. PIM2 phosphorylates tumor suppressor tuberculous sclerosis complex-2 (TSC2) causing unleash of mTORC1 signaling from TSC2 repression, which results in cell proliferation [11]. RSK2, a critical serine/threonine-protein kinase that acts downstream of ERK in FLT3-ITD-acute myeloid leukemia (AML), was also identified as another PIM2 target [12]. Apoptosis inhibitor 5 (API-5) could be phosphorylated by PIM2 leading to liver tumor progression [13]. Co-expression of PIM-2 and c-Myc transgenes induces malignant transformation [2]. Several studies demonstrated that PIM2 dysregulation was associated with several cancers, *e.g*., lymphoma [14], leukemia [15], multiple myeloma [16], prostate cancer [17,18], hepatocellular carcinoma [19]. Overexpression of *pim2* was linked to poor survival of AML patients [20]. PIM kinases have been found to overexpress and play a vital role in the regulation of different proteins responsible for ovarian cancer tumorigenesis [21]. These data indicate that PIM2 kinase is a potential therapeutic target for pan anti-cancer *via* restoration of apoptosis in drug resistant cancer cases, particularly hematopoietic malignancies and some solid cancers.

Antibodies have been used for treatment and intervention of human diseases, both infectious and non-infectious [22]. For safety issue, the therapeutic antibodies should have negligible or no immunogenicity in the recipients, especially for cancers which requires repeated dosages in long-term therapeutic protocol; implying that fully human antibodies should be the safest antibody isotype/format; in opposition to the animal derived counterparts, such as mouse monoclonal or even humanized-animal antibodies. Nevertheless, production of fully human antibody isotype that target proteins of human own species, *e.g*., human oncoproteins overexpressed by cancer cells, is relatively difficult and requires a particular strategy (B cells in the peripheral lymphoid tissues have passed bone marrow selection and not supposed to have B cell receptors to own antigens). In this study, engineered fully human single-chain antibody variable fragments (HuscFvs) that binds to human PIM2 at the critical kinase residues are generated *in vitro*. They should be tested further step-by-step towards a clinical use as an adjunctive therapeutic against cancers *via* PIM2 kinase inhibition.

## 2. Results

### 2.1. Expressions of Pim2 by Normal Blood Cell Subpopulations and Cancer Cells

**Figure 1:**
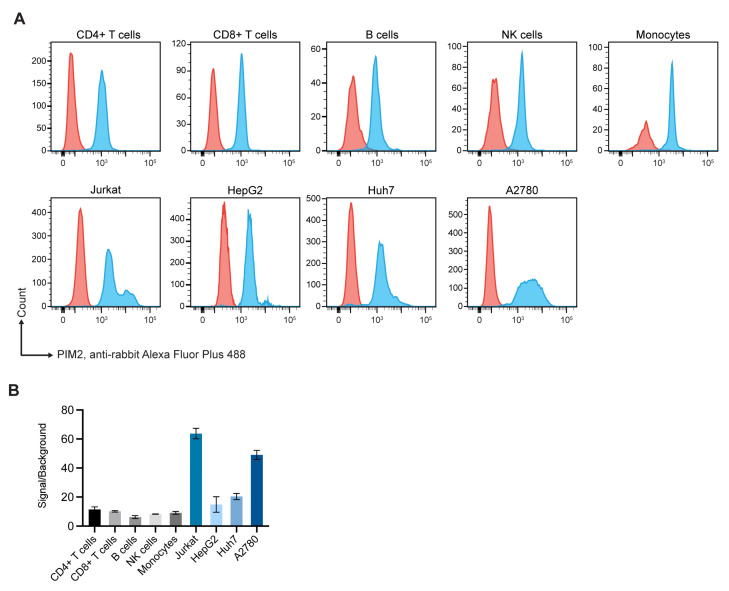
Flow cytometric analysis of PIM2 expression by normal blood cells and cancer cells. (**A**) PIM2 expression by sub-populations of peripheral blood cells of healthy donor and some cancer cells (cyan histograms). Controls were cells stained with conjugate only (orange). Upper panels are various sub-populations of one healthy donor (as representative) including CD4^+^ T cells, CD8^+^ T cells, B cells, NK cells and monocytes; lower panels are various cancer cells including Jurkat T cells (human leukemic T cells), HepG2 cells (human liver cancer cells), Huh7 cells (human hepatocarcinoma cells), and A2780 (human ovarian cancer cells). (**B**) Bar charts displaying ratio between geometric mean of cells (three normal donors and cancer cells) stained for PIM2 (signal) and cells stained with conjugate control (background). Results are from replicative experiments.

Flow cytometric analysis revealed that the human cancer cells tested expressed high levels of PIM2, compared to subpopulations of blood cells of three healthy donors (Figure 1). 

### 2.2. Recombinant PIM2

**Figure 2:**
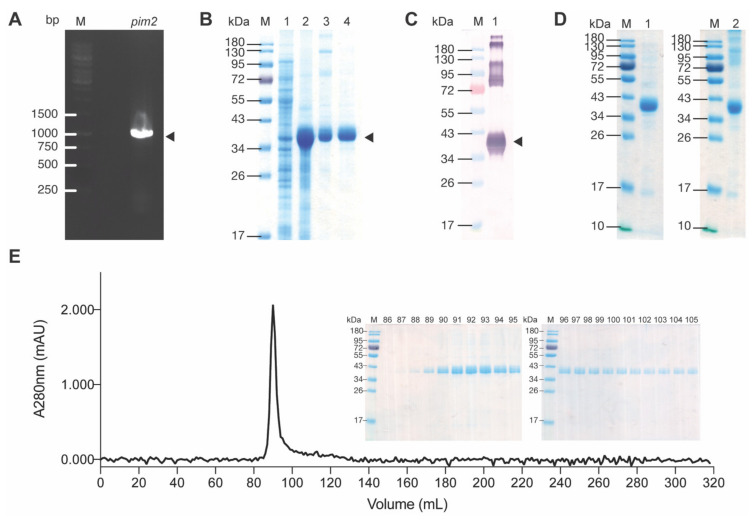
Preparation of recombinant PIM2 (rPIM2). (**A**) PCR amplicon of *pim2* cDNA (~933 bp). Lane M, 1 kb DNA ladder. Numbers at the left are DNA sizes in base pairs (bp). (**B**) SDS-PAGE-separated patterns of CBB-stained-rPIM2; Lane M, PageRuler™ Prestained Protein ladder; Lane 1, lysate of original NiCo21 (DE3) *E. coli* (negative for rPIM2); Lane 2, insoluble fraction of pLATE52-*pim2*-transformed-NiCo21 (DE3) *E. coli*; Lane 3, purified inclusion body of the pLATE52-*pim2*-transformed-NiCo21 (DE3) *E. coli*; and Lane 4, purified rPIM2. (**C**) Western blot pattern of purified rPIM2 (Lane 1). Lane M, PageRuler™ Prestained Protein Ladder. (**D**) SDS-PAGE- and native-PAGE-separated rPIM2 stained by CBB; Lanes M, PageRuler™ Prestained Protein ladder; Lane 1, refolded purified rPIM2 (1 µg) in SDS-PAGE gel; and Lane 2, rPIM2 in native-PAGE. (**E**) Chromatogram of rPIM2 (15 mg) separated on Sephacryl-200 column chromatography. Inset in (**E**) are SDS-GAGE-patterns of proteins in the fractions 86 to 105 stained by CBB; the protein in the representative band was identified as PIM2 by LC-MS/MS. Numbers at the left of (**B**), (**C**), (**D**) and (**E inset**) are protein molecular masses in kDa.

The PCR amplicon of *pim2* using Jurkat cell complementary DNA (cDNA) as template revealed DNA band at ~933 bp (Figure 2A). The DNA was cloned into pLATE52 vector and the recombinant *pLATE52-pim2* plasmid was put into NiCo21 (DE3) *E. coli.* After growing the transformed *E. coli* in isopropyl β-d-1-thiogalactopyranoside (IPTG)-induced medium, the bacterial lysate was found to contain the recombinant protein at ~37–40 kDa as revealed by SDS-PAGE and Coomassie Brilliant Blue G-250 (CBB) staining (Figure 2B) and Western blotting probed with mouse anti-His antibody (Figure 2C). Mass spectrometry verified that the recombinant protein was human PIM2 (data not shown). From 250 mL of transformed NiCo21 (DE3) *E. coli* culture, 312 mg of wet inclusion body (IB) were isolated. Total protein content of the purified IB determined by BCA method was 34.72 mg. The IB (20 mg) was re-solubilized. After refolding dialysis, 18.4 mg of proteins were recovered. Figure 2D shows rPIM2 separated by SDS-PAGE and native-PAGE after CBB staining. Size exclusion column chromatography (SEC) of the refolded PIM2 on Sephacryl-200 revealed one discrete protein peak (Figure 2E).

### 2.3. Production of HuscFvs to Recombinant PIM2 (rPIM2) and Binding of the HuscFvs to rPIM2 and Native PIM2

**Figure 3:**
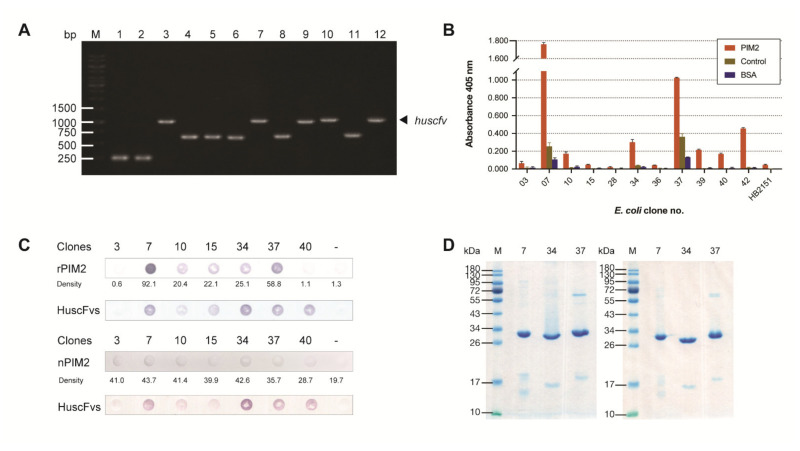
Preparation of HuscFvs that bound to rPIM2. (**A**) Examples of PCR amplicons of *huscfvs* (~1000 bp, arrow) in different phage-transformed-HB2151 *E. coli* clones. Lane M, 1 kb DNA ladder; numbers at the left are DNA sizes in bp. (**B**) Indirect ELISA for testing binding of HuscFvs in lysates of different phage-transformed *E. coli* clones to rPIM2 using control antigens, i.e., unrelated His-tagged protein (control) and BSA, and lysate of original HB2151 *E. coli* (HB2151) as background binding control (negative HuscFv). Lysates of the 11 *E. coli* clones (Nos. 3, 7, 10, 15, 28, 34, 36, 37, 39, 40 and 42) showed OD 405 nm to rPIM2:OD 405 nm to BSA greater than two. After DNA sequencing, *huscfvs* of Clones 15, 36 and 39 were sibling clones; therefore, only lysates of Clones 3, 7, 10, 15, 28, 34, 37, 40 and 42 were tested for binding to rPIM2 and native PIM2 (nPIM2) from cancer cell lysate by using combined co-immunoprecipitation (Co-IP) and dot-ELISA. (**C**) Combined Co-IP and dot ELISA for testing binding of the HuscFvs of Clones 3, 7, 10, 15, 34, 37 and 40 to rPIM2 and nPIM2 in lysate of Jurkat cells. In this experiment, the strep tagged-HuscFvs of individual *E. coli* clones were immobilized on MagStrep “Type 3” XT beads and the HuscFvs-coated beads were incubated with either rPIM2 or lysate of Jurkat cells containing nPIM2; thereafter, the bead-bound substances were eluted and dotted onto nitrocellulose strips; dot-ELISA was performed to detect rPIM2/nPIM2 and HuscFvs in the same bead-eluates. The densities of PIM2 in individual dots are shown under the PIM2 strips. The dot-ELISA was used instead of Western blotting as there were only minute amounts of the target reactants in the eluted samples. Symbol “-“ indicates eluate of the bead incubated with buffer alone (negative control). (**D**) Patterns of CBB-stained purified HuscFv7, HuscFv34 and HuscFv37 after SDS-PAGE (left panel) and native-PAGE (right panel).

Phage clones of the HuscFv phage display library [23] that bound to the rPIM2 in the phage bio-panning process were used to infect non-suppressor HB2151 *E. coli*. From 48 single colonies of phage-transformed-HB2151 *E. coli* that grew on the selective agar plates, 26 colonies carried *huscfvs*, which appeared as PCR amplicons at ~1000 bp (Figure 3A). The *huscfv*-positive *E. coli* clones were grown in IPTG-conditioned medium. The HuscFvs in their lysates were tested for binding to rPIM2 by indirect ELISA using unrelated (His-tagged) protein and BSA as control antigens, and lysate of original HB2151 *E. coli* (HB2151) as background binding control. Lysates of 11 clones (Nos. 3, 7, 10, 15, 28, 34, 36, 37, 39, 40 and 42) showed OD 405 nm to rPIM2:OD 405 nm to BSA greater than 2 (Figure 3B). From DNA sequencing of their *huscfvs*, Clones 15, 36 and 39 were sibling clones; therefore, only Clones 3, 7, 10, 15, 28, 34, 37, 40 and 42 were tested further. 

The homology of the amino acid sequences of HuscFvs deduced from the *huscfvs* of the *E. coli* Clones 3, 7, 10, 15, 28, 34, 37, 40 and 42 compared with the closest human V framework regions (FRs) ranged from 88 to 100%, indicating that the HuscFvs are human immunoglobulins (Table 1).

The *huscfv* sequences of the HB2151 *E. coli* Clones 3, 7, 10, 15, 28, 34, 37, 40 and 42 were subcloned to pET24DS, which contained gene encoding signal peptide, and the recombinant plasmids were introduced to NiCo21 (DE3) *E. coli* expression host. After this subcloning, the transformed-NiCo21 (DE3) *E. coli* Clones 28 and 42 did not express HuscFvs in small-scale expression. The HuscFvs in periplasms of *E. coli* Clones 3, 7, 10, 15, 34, 37 and 40 were retested for binding to rPIM2 and native PIM2 in Jurkat cell lysate by using combined co-immunoprecipitation (Co-IP) and dot-ELISA (Figure 3C). Three *E. coli* clones (7, 34 and 37) which expressed sufficient amounts of the respective HuscFvs, showed high ELISA signals and bound to both rPIM2 and nPIM2 in dot-ELISA were studied further. 

The HuscFvs from the three *E. coli* clones were subjected to large-scale expression. The yields of the soluble HuscFvs isolated from the periplasms of 1 L culture of the transformed NiCo21 (DE3) *E. coli* ranged from 468 to 1450 μg. Patterns of SDS-PAGE- and native-PAGE-separated purified HuscFvs 7, 34 and 37 after CBB-staining are shown in Figure 3D.

### 2.4. Computerized Simulation for Determining Presumptive Region(s) and Residues of PIM2 That Were Bound by the HuscFvs 

**Figure 4:**
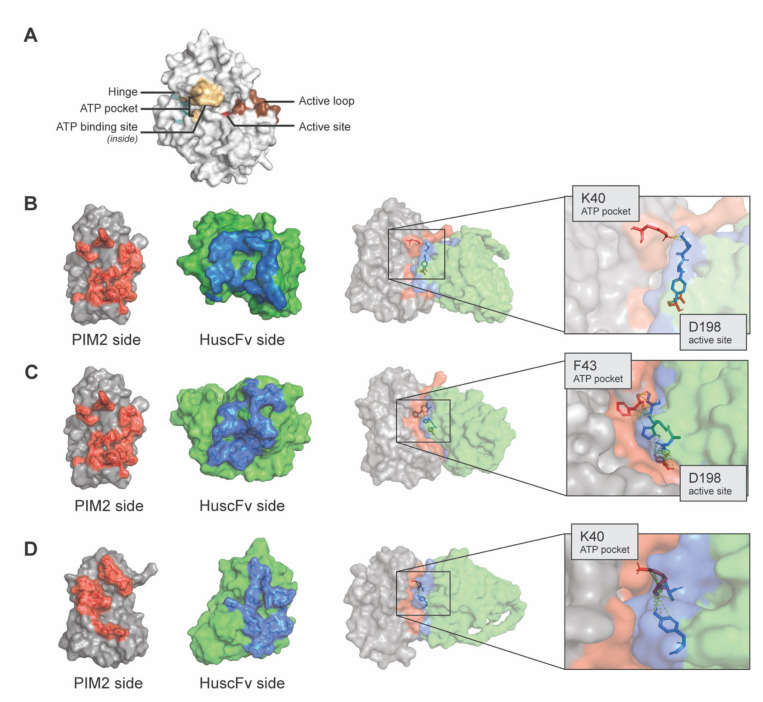
Computerized simulation for determining interaction of the HuscFvs with PIM2. (**A**) Three-dimensional model of PIM2 showing ATP pocket (orange), active loop (brown) and the active site within the active loop (red). (**B**–**D**) Interaction of HuscFv7, HuscFv34 and HuscFv37 to PIM2, respectively.

The PIM2 residues presumptively formed contact interface with the HuscFv7, HuscFv34 and HuscFv37 revealed by the computerized simulation are shown in Figure 4. The results of the *in-silico* analysis showed that the HuscFvs of the three *E. coli* clones presumptively interacted with residues that actively involved in the PIM2 kinase activity including K40 and/or F43 located in the ATP pocket, and D198 which is the residue stabilizing a constitutively active loop conformation of PIM2 kinase. Table 2 gives details on the residues and site(s) of PIM2 that formed contact interface with the respective HuscFvs.

### 2.5. Effective Concentration 50 (EC50) of HuscFvs and HuscFv-mediated Inhibition of PIM2 Kinase Activity

**Figure 5:**
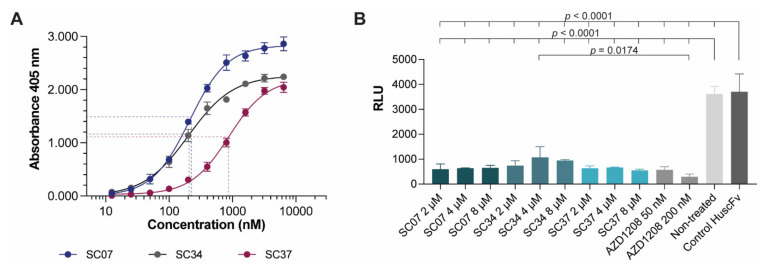
Effective concentration 50 (EC50) of HuscFvs and PIM2 kinase inhibition assay. (**A**) Effective concentration 50 (EC50) of purified HuscFv7 (SC07), HuscFv34 (SC34) and HuscFV34 (SC34). Each point represents mean of three individual data and error bars represent standard deviation of the dataset. (**B**) PIM2 kinase inhibition assay of HuscFv7 (SC07), HuscFv34 (SC34), and HuscFv37 (SC37). The experiment without treatment (buffer) was included and represented the system control. Control HuscFv at 8 µM was included as the negative control. AZD1208 at 50 and 200 nM were used as positive kinase inhibitor controls. Each point except non-treated and control HuscFv represented three individual datasets. Non-treated and control HuscFv represented two individual data. Error bar represents standard deviation of individual dataset.

Effective concentrations of the HuscFvs of the selected *E. coli* Clones 7, 34 and 37 (HuscFv7, HuscFv34 and HuscFv37, respectively) were determined by indirect ELISA. The calculated effective concentration 50 (EC50) of the HuscFv7, HuscFv34 and HuscFv37 were 211.7, 202.5, and 878.3 nM, respectively (Figure 5A).

The PIM2 kinase inhibitory activity of the HuscFvs were determined using a small chemical, AZD1208, which is an ATP competitive inhibitor of all PIM isoforms (pan-PIM inhibitor) as positive kinase inhibition control. Control HuscFv and non-treatment control (buffer alone) served as negative inhibition controls. Principles of the PIM2 kinase and PIM2 kinase inhibition assays are illustrated in Supplementary Figure S1. The results in Figure 5B illustrate the significant PIM inhibitory activity of HuscFv7, HuscFv34 and HuscFv47 at all concentrations tested (2, 4 and 8 μM) and both concentrations of the AZD1208 (50 and 200 nM), compared to the control HuscFv and non-treatment control (*p* < 0.0001). The inhibitory activities of the HuscFvs of all clones were not different (*p* > 0.05), and were, more or less, similar to the AZD activity (*p* > 0.05). The relative luminescence units (RLU) of the non-treated and the control HuscFv were not different (*p* > 0.05). Data are from one of the two replicative experiments.

## 3. Discussion

PIM2 kinase plays an important role in tumorigenesis of many cancers, including leukemia, multiple myeloma and solid tumors, *e.g*., lymphomas, ovarian, prostate, breast, stomach, liver, and lung cancers [24]. PIM2 kinase activates transcription of genes involved in cell survival, cell proliferation, and cell-cycle progression *via* multiple downstream signaling molecules [24]. High levels of PIM2 kinase are expressed by different cancer cells, as verified by our results in Figure 1, and this has been associated with poor prognosis of cancer patients [24]. Thus, PIM2 kinase is a rational target of pan anti-cancer therapeutics [6,25]. Several chemical inhibitors targeting PIMs/PIM2 or their downstream signaling molecules have been developed for treatment of a variety of cancers. For examples, thiazolidine-2,4-dione-family compounds (SMI-4a and SMI-16a) [26,27], DHPCC-9 [28], SGI-1776 [29,30,31], AZD1208 [32], CX-6258 or Compound 13 [33], JP11646 [34], PIM447 (LGH447 dihydrochloride) [35], imidazopyridazine-thiazolidinediones with YPC-21440 and/or YPC-21817 [36] and others. While these chemical drugs are highly effective against PIM kinase activity *in vitro* or in animal model of cancer, their off-target toxicity is common in clinical trials in oncology, so that they could not be advanced to official approval for clinical application [37]. Development of anti-PIMs/PIM2 agents that are biocompatible to humans for long-term treatment of cancers through repeated administration is rational. In this study, we offer another ramification of the PIM kinase inhibition, i.e., fully human single-chain antibodies directed to the residues critical for the PIMs/PIM2 kinase activity.

Nowadays, fully human antibody molecules can be produced by using several strategies including immunization of animals (rodent, cattle) that carried human immunoglobulin transgenes, with the target antigens [38]. Subsequently, polyclonal human antibodies could be obtained from the serum of immunized transgenic large animals, such as cattle. Nevertheless, the immune serum also contains non-specific immunoglobulins and other serum proteins. The conventional hybridoma technology can be used to generate rodent B lymphocyte-myeloma hybrid cells that secrete monoclonal antibodies. Alternatively, human monoclonal antibodies can be produced by immune B cell clones that are immortalized by Epstein–Barr virus transformation [39]. Besides, human full-length monoclonal antibodies or antibody fragments can be produced *in vitro* from antibody display libraries expressed by prokaryotic or eukaryotic cells [40,41,42]. However, the human antibodies produced by these methods are directed mainly against foreign substances and hardly human antibodies to human own proteins, such as those highly expressed by cancer cells. Thus, most therapeutic antibodies for cancer immunotherapy are in humanized format [22,43]. 

In 2009, we constructed a human scFv phage display library [23]. The pool of human cDNA was used as templates for amplification of *vh* and *vl* sequences by error prone-PCR using human degenerate primers designed from all families/subfamilies of human immunoglobulin genes. The combinatorial paired degenerate primers and relatively less stringent *Taq* DNA polymerase were used such that one cDNA template could give rise to different PCR amplified products. By this strategy, not only the diversity of the antibody gene repertoire was increased, but also by chances, some of them happened to be similar to the immunoglobulin genes before B cell selection (germline immunoglobulin genes) in the bone marrow; hence, a portion of phages displaying HuscFvs in the library carry HuscFvs that bind to human components (autogens). 

The *E. coli* derived-recombinant human PIM2 (rPIM2) was produced as inclusion bodies that required purification and refolding. The majority of the purified rPIM2 after refolding migrated similarly in both SDS-PAGE and native-PAGE as predominant protein bands at approximately 37–40 kDa; indicating that most of the rPIM2 in the preparation is monomeric. This was verified by size exclusion column chromatography on the Sephacryl-200 resin column loaded with the rPIM2 that revealed one discrete protein peak in the column eluted fractions. Thus, the rPIM2 preparation was used as an antigenic bait to fish out the phage clones displaying HuscFvs that bound to the antigen from the HuscFv phage display library by bio-panning. HuscFvs of three selected *E. coli* clones (Clones 7, 34 and 37) bound to both recombinant and native PIM2. These *E. coli* clones could produce the HuscFvs in sufficient amounts; thus, their HuscFvs were studied further. Amino acid sequences of the immunoglobulin framework regions of the rPIM2-bound HuscFvs of Clones 7, 34 and 37 (HuscFv7, HuscFv34 and HuscFv37) show 88–100% homology with those of human immunoglobulins of the database (Table 2), verifying that they are fully human proteins. Therefore, these HuscFvs should have negligible (if there were any) immunogenicity when used in the repeated treatment of patients with human cancers in the future, and thus they should be safe. *In silico* analysis of binding mode of the HuscFvs and PIM2 kinase revealed that the HuscFv7 (*via* VH-CDR3), HuscFv34 (*via* VH-CDR2) and HuscFv37 (*via* VH-CDR3 and VL-CDR2) presumptively interacted by hydrogen bonding with K40 and/or F43 located in the ATP pocket of the PIM2 kinase. The VH-CDR3 of HuscFv7 and VL-CDR2 of HuscFv34 also form hydrogen bonding with D198 which is the residue stabilizing a constitutively active loop conformation of PIM2 kinase. 

The selected PIM2-bound HuscFv7, HuscFv34 and HuscFv37 had the EC50 in the nanomolar range. They effectively inhibited the PIM2 kinase activity at the concentrations used in the experiments (2, 4 and 8 μM) and they were as effective as the AZD1208, the small chemical PIM kinase inhibitor (not for *in vivo* use). The dose-dependent inhibitory activities were not seen at the concentrations tested, probably due to the saturated amounts of the inhibitors that were used. Unfortunately, smaller amounts were not tested.

PIMs are located intracellularly, and usually cannot be accessed by conventional four-chain antibodies, single-chain antibodies or nanobodies (single-domain antibodies). Nevertheless, the antibodies, particularly the single-chain antibodies or nanobodies can be engineered into a safe cell-penetrable format for their accessibility to the intracellular target, i.e., by molecularly linking them to a human cell-penetrating peptide (CPP, which is a short peptide that can carry various types of cargo molecules across the formidable plasma membranes into cells) such as AA3H peptide (ASIWVGHRG) derived from human annexin III [44] or ECP^32–41^, derived from the core heparin-binding motif of human eosinophil cationic protein (ECP) [45] or other non-immunogenic CPP such as nonaarginine (R9) [46]. Alternatively, the antibodies can be entrapped into suitable biocompatible nanoparticles that can traverse across the plasma membrane [47]. The fully human single-chain antibodies produced in this study have high potential for developing and testing further towards a clinical use as a safe PIM inhibitor for pan-immunotherapy of human cancers.

## 4. Materials and Methods

### 4.1. Verification of PIM2 Upregulation in Cancer Cells 

Cancer cell lines used in this study were Jurkat T cells (immortalized leukemic T lymphocytes), HepG2 and Huh7 (human hepatocellular carcinoma cells), and A2780 (human ovarian cancer cells; provided by Dr. Somponnat Sampattavanich, Department of Pharmacology, Faculty of Medicine Siriraj Hospital, Mahidol University, Bangkok). The Jurkat and A2780 cells were cultured in RPMI-1640 (Gibco, Thermo Fisher Scientific, Waltham, MA, USA) supplemented with 1× penicillin-streptomycin (Corning, NY, USA) and 2 mM GlutaGro™ (Corning) (complete RPMI medium). The HepG2 and Huh7 cells were cultured and maintained in Dulbecco’s Modified Eagle’s Medium (DMEM) (Gibco) supplemented similarly to the complete RPMI-1640 medium (complete DMEM). 

Peripheral blood mononuclear cells (PBMCs) were isolated from blood samples of three healthy volunteers by density gradient centrifugation using Ficoll–Paque (Cytiva, Marlborough, MA, USA). The buffy coat of each blood sample was collected and washed with Dulbecco phosphate buffered saline (DPBS; Gibco). Sub-populations of PBMCs were differentiated by surface staining. The PBMCs were blocked with 10% AB serum and added with PerCP-anti-CD3 (#344814, Biolegend, San Diego, CA, USA), PE-Cyanine7-anti-CD4 (#25-0047-42, eBioscience, Thermo Fisher Scientific), PE/Dazzle™ 594-anti-CD8 (#344744, Biolegend), and AlexaFluor 647-anti-CD22 (#302517, Biolegend). After keeping at room temperature for 30 min, the cells were washed and subsequently stained for intracellular PIM2 expression. Experiment involved human samples were approved by Institutional Review Board (IRB) of the Faculty of Medicine Siriraj Hospital, Mahidol University (no. Si651/2018).

Expression of PIM2 in the cancer cells were determined by flow cytometric analysis in comparison to blood cell subpopulations of normal healthy subjects. Log-phase grown cancer cells were washed with DPBS, fixed and permeabilized with 4% paraformaldehyde and 1× intracellular staining permeabilization wash buffer (Biolegend). The cells were blocked with 10% AB serum, washed, and added with monoclonal anti-rPIM2 (RabMab; ab129193; Abcam, Cambridge, MA, USA). After keeping at room temperature for 30 min, the cells were washed, and added with AlexaFlour Plus488-goat anti-rabbit isotype (A32731; Invitrogen, Thermo Fisher Scientific) for 30 min. Controls included cells incubated with AlexaFlour Plus488-goat anti-rabbit isotype (conjugate). The cells of all preparations were washed, re-suspended in flow cytometry staining buffer, and subjected to flow cytometric analysis (LSRFortessa, BD, Franklin Lakes, NJ, USA) for determining their PIM2 expression.

### 4.2. Preparation of Recombinant PIM2 (rPIM2)

Total RNA was extracted from log phase grown-Jurkat T cells (5 × 10^6^ cells) by using blood/cell total RNA mini kit (RB100, Geneaid, New Taipei, Taiwan). Quality of the total RNA was determined by using NanoDrop™ 2000 (Thermo Fisher Scientific) at 260/280 nm. The good quality RNA (260/280 nm was higher than 1.8) was used as template for cDNA synthesis based on the protocol of RevertAid first strand cDNA synthesis kit (Thermo Fisher Scientific). The *pim2* cDNA was amplified by PCR using hi-fidelity Phusion polymerase (Thermo Fisher Scientific) and synthesized primers specific to *pim2*, i.e., forward primer: 5′-TTGACCAAGCCTCTACAGGGGCC-3′ and reverse primer: 5′-GGGTAGCAAGGACCAGGCCAAAG-3′ (IDT, Coralville, IA, USA). The PCR reaction mixture contained 10 ng cDNA, 1× Phusion HF buffer, 200 µM each dNTPs, 0.5 µM each forward and reverse primers, 3% (*v*/*v*) dimethyl sulfoxide, and 0.02 U/µL Phusion DNA polymerase. The PCR thermal cycling condition included initial denaturation at 98 °C, 30 s; 35 cycles of denaturation at 98 °C for 10 s, annealing at 58 °C for 30 s and extension at 72 °C for 30 s; and final extension at 72 °C for 5 min. The PCR product (50 ng) was then cloned into pJET1.2 vector using CloneJet PCR cloning kit (Thermo Fisher Scientific), and the recombinant vector was transformed into JM109 *E. coli* using TransformAid bacterial transformation kit (Thermo Fisher Scientific). The bacteria were spread onto Luria-Bertani (LB)-agar plate supplemented with 100 µg/mL ampicillin (GDH, Bangkok, Thailand) (LB-A) agar. Plasmids containing *pim2* cDNA was then extracted from the 5-mL overnight-grown JM109 *E. coli* in LB broth at 37 °C with shaking at 250 rpm, and quantified by NanoDrop, sequenced by Sanger method (1st BASE, Selangor, Malaysia) and aligned with *pim2* of the database (Accession no. AK290931.1) in CLC Genomic Workbench 12 (Qiagen, Hilden, Germany). The verified *pim2* cDNA was subcloned into pLATE52 expression vector by using aLICator ligation-independent cloning and expression system (Thermo Fisher Scientific), and transformed into JM109 *E. coli*. After re-verification of the *pim2* by DNA sequencing (1st BASE, Malaysia), the pLATE52-*pim2* construct was isolated and introduced to NiCo21 (DE3) *E. coli* expression host. The transformed NiCo21 (DE3) *E. coli* was cultured in 250 mL 2× YT broth containing 100 µg/mL ampicillin and 2% (*w*/*v*) D-glucose (Kemaus, CherryBrook, New South Wales, Australia) (2× YT-AG) at 37 °C with shaking aeration at 250 rpm until the culture reached OD 600 nm at 0.5. Then, the culture medium was replaced by 2× YT containing 100 µg/mL ampicillin and 0.1 mM isopropyl β-d-1-thiogalactopyranoside (IPTG, Vivantis, Selangor, Malaysia) and cultured at 30 °C with shaking at 250 rpm for additional 5 h for recombinant protein expression. 

The bacterial inclusion body containing the recombinant protein was purified by non-chromatographic purification. First, *E. coli* cells were harvested by centrifugation (4000× *g*, 15 min). The bacterial cells in each gram of the bacterial paste were lysed in 5 mL BugBuster^®^ protein extraction reagent (Millipore, Merck KGaA, Darmstadt, Germany) and 10 µL Lysonase (Millipore, Merck KGaA). After centrifugation, inclusion body in the pellet was washed with 10 mL of wash-100 buffer [phosphate buffer, pH 8.0; 500 mM sodium chloride (Kemaus); 5 mM ethylenediaminetetraacetic acid (Kemaus); and 1% (*v*/*v*) Triton X-100 (Affymetrix, Thermo Fisher Scientific)], 10 mL of wash-buffer-114 [phosphate buffer, pH 8.0; 50 mM sodium chloride; and 1% (*v*/*v*) Triton X-114 (Sigma, Merck KGaA)], and 10 mL deionized distilled water, respectively. The purified inclusion body (0.5 mg) was then solubilized in 1 mL solubilization buffer [50 mM CAPS, pH 11.0 (Sigma, Merck KGaA) supplemented with 0.3% (*w*/*v*) sodium lauroyl sarcosinate (sarkosyl, Sigma, Merck KGaA) and 1 mM dithiothreitol (DTT, Affymetrix)]. After removing insolubilized part by centrifugation (10,000× *g*, 4 °C, 10 min), the solubilized recombinant protein was refolded in 20 mM Tris pH 8.5 with and without 0.1 mM DTT, respectively. The refolded rPIM2 was subjected to SDS-PAGE, native-PAGE and protein staining using Coomassie Brilliant Blue G-250 dye (CBB), Western blot analysis, and size exclusion chromatography (SEC). Refolded rPIM2 was supplemented with 60 mM Trehalose and stored at −80 °C for further use.

### 4.3. SDS-PAGE, Native-PAGE and Western Blot Analysis

Discontinuous SDS-polyacrylamide gels and native-polyacrylamide gels were cast in Mini-PROTEAN^®^ Tetra Handcast Systems (Bio-Rad, Hercules, CA, USA). The samples were mixed with either 6× loading buffer or 6× native loading buffer. For SDS-PAGE, the samples were heated at 95 °C. Samples and protein marker were loaded into designated wells of the cast gel. The gels were electrophoresed under 20 mA current per gel in electrode buffer until the font dye reached lower edge of the gel. CBB staining was performed by submerging the gel into 20 mL Quick Coomassie Stain (Protein Ark, Sheffield, UK).

Western blotting was performed by transferring the separated proteins in the gels onto 45 µm-nitrocellulose membranes (NC) (Cytiva) under 100 V power for 1 h. The unoccupied sites on the blotted NC were blocked by blocking agents, *e.g.*, 3% skim milk in TBS-T, 5% bovine serum albumin, or protein-free blocking buffer (Pierce™ Protein-Free (TBST) Blocking Buffer, Thermo Fisher Scientific). The membranes were subsequently probed with 1:3000 mouse anti-His tag primary antibody (Bio-Rad) in 5 mL TBS-T. After allowing primary antibody to bind to the target for 1 h, the membranes were washed thoroughly by TBS-T followed by adding with 1:3000 horseradish peroxidase (HRP)-conjugated goat anti-mouse immunoglobulin (SouthernBiotech, Birmingham, AL, USA) in 5 mL TBS-T for 1 h and the membranes were washed. The color was developed by adding BCIP/NBT (KPL, SeraCare, Milford, MA, USA) to the Tris-HCl, pH 9.6 pre-equilibrated membranes.

### 4.4. Size Exclusion Column Chromatography (SEC)

The rPIM2 was subjected to size exclusion column chromatography (SEC). Fifteen milligrams of purified and refolded rPIM2 was loaded onto Sephacryl-200 HR 26/60 column (Cytiva). One column volume (CV) of the running buffer (50 mM Tris and 150 mM sodium chloride, pH 7.2) was then pumped into the column. One milliliter-fractions of the eluates were collected. Then, 280 nm absorbance of each fraction was measured using Nanodrop™ 8000 (Thermo Fisher Scientific). The chromatogram was generated by plotting elution volume (mL) against A280nm using Prism 9.2 (Graphpad, San Diego, CA, USA). Proteins in the fractions with detectable A280nm were subjected to SDS-PAGE and stained by CBB; the representative protein band was excised and identified by LC-MS/MS.

### 4.5. HuscFv Phage Display Library

The human scFv (HuscFv) phage display library used in this study was constructed previously [23]. Peripheral blood mononuclear cells (PBMCs) obtained from 50 young adult volunteers (25 male and 25 female) and 10 buffy coat preparations obtained from the Thai Red Cross blood bank were used as a source of immunoglobulin genes. Total RNA was extracted from the cells of each subject/buffy coat and pooled. Complementary DNAs were synthesized using the RNA pool as a template. Genes coding for all families and subfamilies of human immunoglobulin variable fragments (VH and VL domains) were amplified using 42 pairs of degenerate oligonucleotide primers to *vh* (16 forward and 3 reverse primers) and 26 pairs of degenerate primers to *vl* (13 forward and 2 reverse primers), such that one cDNA template can yield multiple amplicon variants (i.e., increased antibody gene diversity and the possibility of obtaining also gene sequences that encode self-antigens that are lacking in the normal peripheral blood B cell pool). The VH and VL genes (*vh* and v*l* sequences) were linked randomly [*via* a polynucleotide linker coding for (Gly_4_Ser_1_)_3_] to yield a repertoire of genes coding for single-chain variable antibody fragments (*vh*-linker *vl* or *scfvs*). The *scfv* repertoire was cloned into pCANTAB 5E phagemid downstream of the gene coding for the phage coat protein, P3. The recombinant phagemids were introduced into competent TG1 *E. coli*. The transformed *E. coli* were grown and co-infected with helper phage, M13KO7. The complete phage particles displaying the human scFvs (HuscFvs) as fusion partners of the P3 protein and contained the respective HuscFv genes (*huscfvs*) in the phage genomes were recovered from the *E. coli* culture supernatant (HuscFv phage display library was obtained). The HuscFvs-diversity of this library was approximately 2.6 × 10^8^ [23]. After one cycle of library propagation in TG1 *E. coli*, approximately 2.6 × 10^12^ cfu/mL of complete phage particles were obtained [23].

### 4.6. Production of HuscFvs to rPIM2

For selection of phage clones displaying HuscFvs that bound to rPIM2 from the HuscFv phage display library, the phage bio-panning was performed using rPIM2 as the panning antigen. Recombinant PIM2 [500 ng in 100 μL phosphate buffered saline, pH 7.4 (PBS)] was added into a well of a 96-well-microplate and kept at 37 °C overnight. Recombinant PIM2-coated well was washed three times with PBS containing 0.05% Tween-20 (PBS-T), blocked with protein-free blocking solution [Pierce™ Protein-Free (PBS) Blocking Buffer, Thermo Fisher Scientific] for 1 h, washed again with PBS-T, and added with the 50 μL of HuscFv phage display library. After keeping at room temperature (25 °C) for 1 h, the fluid containing unbound phages was removed; the well was washed thoroughly with PBS containing 0.5% (*v*/*v*) Tween-20 before adding with 100 µL mid-log phase grown-HB2151 *E. coli* [K12 Δ(*lac-pro*), *ara*, *nal^r^*, *thi*/F’[*proAB*, *lacI^q^*, *lacZ*Δ*M15*]; lifescience-market.com] and phage transfection was allowed for 30 min. The phage-infected bacteria were spread on 2× YT-AG agar plates and incubated at 37 °C overnight. Single bacterial colonies were screened for recombinant pCANTAB 5E phagemids with inserted *huscfvs* by direct colony PCR, using the phagemid specific primers [23]. The PCR cycling steps included initial denaturation at 95 °C, 10 min; 35 cycles of denaturation at 95 °C for 30 s, annealing at 55 °C for 30 s and extension at 72 °C for 1 min; followed by final extension at 72 °C for 10 min. The HB2151 *E. coli* clones positive for the *huscfvs* were grown in 5-mL auto-induction medium [2× YT, 90 mM potassium phosphate buffer, pH 7.6; 2 mM magnesium sulfate; 0.5% (*w*/*v*) D-glucose; and 0.2% lactose] containing 100 µg/mL ampicillin. Bacterial cells harvested from the cultures were lysed by using 0.5 mL BugBuster^®^ solution (Merck KGaA) supplemented with 25 U/mL Benzonase^®^ (Merck KGaA) and 1:200 protease inhibitor cocktail set III (Merck KGaA). The bacterial lysates were collected after centrifugation (15,000 ×*g*, 4 °C, 15 min). 

Soluble HuscFvs in the *E. coli* lysates were tested for binding to rPIM2 by indirect ELISA [23]. Recombinant PIM2 and control antigens (His-tagged protein and BSA) (100 ng in 100 µL PBS) were added to wells of an ELISA plate and kept at 4 °C overnight. After washing with Tris buffered saline containing 0.1% (*v*/*v*) Tween-20 (TBS-T) and blocking with 5% (*w*/*v*) skim milk, 100 µL of individual *E. coli* lysates were added to appropriate rPIM2 and control antigen coated wells for 1 h. After washing with TBS-T, wells were added with rabbit anti-E tag (1:3000 dilution, ab3397, Abcam) to detect HuscFvs, for 1 h. The signal was developed by adding 1:3000 diluted HRP-conjugated goat anti-rabbit isotype (SouthernBiotech) for 1 h followed by ABTS substrate (KPL, SeraCare) for 30 min with three times TBS-T washing between the steps. The HB2151 *E. coli* clones that the HuscFvs in their lysates gave OD 405 nm to rPIM2 at least 2 times higher than the same lysate to control antigens, were selected for further experiments.

The selected *E. coli* clones were grown in 2× YT-AG broth at 37 °C with shaking at 250 rpm overnight. The *huscfv*-phagemids they carried were isolated using Presto™ mini plasmid kit (RB100, GeneAid) and the *huscfvs* were sequenced (1st BASE). The deduced amino acid sequences of all *huscfvs* were then aligned with human VH and VL sequences of the International Immunogenetics Information System database for verification of their human isotype. The immunoglobulin framework regions (FRs) and the complementarity determining regions (CDRs) of the individual HuscFv sequences were predicted using Pyigclassify [48].

### 4.7. Binding of the HuscFvs to Recombinant and Native PIM2

HuscFvs in NiCo21 (DE3) *E. coli* periplasmic preparations were retested for binding to rPIM2 and native PIM2 in lysate of Jurkat cancer cells by combined co-immunoprecipitation and dot-ELISA. Jurkat cells (10^7^ cells) were harvested and lysed using M-PER™ mammalian protein extraction reagent (Thermo Fisher Scientific) supplemented with 25 U/mL Benzonase^®^ (Merck KGaA) and 1:200 protease inhibitor cocktail set III (Merck KGaA). The cancer cell lysate was then collected by centrifugation at 15,000× *g*, 4 °C, 15 min. The strep-tagged-HuscFvs were immobilized on MagStrep “Type 3” XT beads (IBA Life Sciences, Göttingen, Germany). The rPIM2 and Jurkat cancer cell lysate were added to mix with different aliquots of HuscFvs-bound-magnetic beads. After keeping at room temperature on a rotator for 1 h, the beads were collected, washed, and the bead-bound substances were eluted by using 50 mM biotin in 100 mM Tris-HCl, pH 8.0, containing 150 mM NaCl, 1 mM EDTA. The eluates were subjected to dot-ELISA for detecting the Strep-tagged-HuscFvs and the PIM2 (Western blotting was not performed because of the minute quantities of the recovered target reactants). The eluates were dotted onto nitrocellulose (NC) strips (Cytiva) using Bio-Dot^®^ microfiltration apparatus (Bio-Rad). For detection of rPIM2 and nPIM2 in the eluates, the NC strips were blocked with 5% skim milk before incubating with 1:1000 diluted monoclonal anti-rPIM2 (RabMab; ab129193; Abcam) and added with 1:3000 diluted alkaline phosphatase (AP)-conjugated goat-anti-rabbit isotype (SouthernBiotech) and BCIP/NBT substrate (KPL, SeraCare) for color development, with TBS-T washing between the steps. For detecting HuscFvs, the NC strips were blocked with 3% BSA and the traces of biotin in the bacterial eluates/mammalian cell lysates were covered with 1:1000 diluted biotin-blocking solution (IBA Life Sciences). The strips were incubated with 1:4000 diluted AP-conjugated Strep-TactinXT (IBA Life Sciences); BCIP/NBT substrate (KPL, SeraCare) for color development. The strips were scanned by document scanner (Epson, Nagano, Japan). The density of PIM2 was determined in ImageJ (NIH, Bethesda, MD) by converting image to 8-bit greyscale, followed by inverting the image and selecting the dot space for measurement. Three independent experiments were performed; the results from one of the three replicative experiments are presented.

### 4.8. Large Scale Production of Soluble HuscFvs 

The *huscfv* sequences of the selected HB2151 *E. coli* clones that their HuscFvs bound to rPIM2 and nPIM2 were amplified by hi-fidelity Phusion polymerase (Thermo Fisher Scientific) and subcloned to pET24DS [pET-24a^+^ expression vector which contained gene encoding DsbAss signal peptide at the 5′ of the multiple cloning site (MCS)] and introduced to NiCo21 (DE3) *E. coli* expression host using TransformAid Bacterial Transformation Kit (Thermo Fisher Scientific). Transformants were verified by direct colony-PCR. NiCo21 (DE3) *E. coli* clones carrying pET24DS*-huscfvs* were cultured in 5 mL ZYM-802-GSH medium [1% (*w*/*v*) Bacto™ tryptone, 0.5% (*w*/*v*) Bacto™ yeast extract, 0.2× trace elements, 2 mM magnesium sulfate, 0.8% (*v*/*v*) glycerol, 0.02% (*w*/*v*) D-glucose, 25 mM sodium hydrogen phosphate, 25 mM potassium dihydrogen phosphate, 50 mM ammonium chloride, 5 mM sodium sulfate, and 5 mM reduced L-glutathione] under kanamycin selection (ZYM-802-K-GSH) at 25 °C for 24 h. Bacterial cells were harvested by centrifugation and their periplasmic contents were isolated by using TDRE extraction buffer containing 200 mM Tris, 0.025% (*w*/*v*) sodium deoxycholate, 50 mM L-glutamic acid, 50 mM L-arginine and 50 mM sodium chloride; then the preparations were applied to StrepTrap XT column, a Sepharose^®^ resin coated with Strep-Tactin^®^XT [a specifically engineered streptavidin that bound to strep-tag (Trp-Ser-His-Pro-Gln-Phe-Glu-Lys] (Cytiva) for purification of the soluble strep-tagged HuscFvs. The HuscFvs were analyzed by SDS-PAGE and native PAGE as described in Section 4.3. Alkaline phosphatase-conjugated Strep-Tactin^®^XT (IBA Life Sciences) was used to detect the Strep tagged HuscFvs.

### 4.9. Computerized Simulation for Determining Presumptive Residues of PIMs Bound by the HuscFvs to PIM2 

Deduced HuscFv sequences were submitted to I-TASSER for three-dimensional structure (3D) building [49]. The predicted models were refined by using the high-resolution protein structure refinement, ModRefiner [50] and were sequentially refined at atomic level by using Fragment-Guided MD stimulation, FG-MD [51]. The PIM2 crystal structure (Uniprot PDB: 4 × 7Q) and HuscFv models were subjected to ClusPro 2.0 server for protein-antibody interaction [52]. Binding energy between HuscFv and PIM2 were predicted using protein binding energy prediction, PRODIGY [53]. The models with lowest Gibb’s free energy were then chosen for detailed analysis. The interactive surface of HuscFvs and PIM2 was identify by using BindProfX [54]. The protein structure models and the molecular interactions were built and visualized by using the Pymol software (The PyMOL Molecular Graphics System, Schrödinger, LLC, New York, NY, USA).

### 4.10. Determination of Effective Concentration-50 (EC50) of the HuscFvs

One hundred nanograms of rPIM2 were added to individual ELISA wells and kept at 4 °C overnight. After washing three times with TBS-T, 3% (*w*/*v*) BSA was applied to block the remaining empty spaces on the well surface. HuscFvs were diluted two-fold serially (started at 6.4 µM). Varying HuscFv concentrations were added to the rPIM2 coated wells (triplicate) for 1 h and the wells were washed with PBST. Trace of biotin that might be present in the BSA were masked by adding 1:1000 biotin blocking buffer (IBA Life Sciences) for 10 min before adding 1:4000 HRP-conjugated Strep-TactinXT (100 µL) to each well. After 1 h, the wells were washed by TBS-T. Enzyme substrate, i.e., 2,2′-Azino-bis(3-ethylbenzothiazoline-6-sulfonic acid) (ABTS) was used to develop color. The absorbance at 405 nm was measured. The EC50 of the HuscFvs of individual *E. coli* clones were extrapolated from the curves constructed by plotting the HuscFv concentrations (X axis) against the A405nm (Y axis). Three independent experiments were performed

### 4.11. Kinase and Kinase Inhibition Assays

The principle of the PIM kinase assay is the detection of by-product of PIM activity. In the assay, active PIM functions by phosphorylating the S6K substrate using ATP, giving rise to the phosphorylated-S6K and ADP by-product. The ADP-Glo™ reagent was then added to the reaction to deplete remaining ATP in the reaction. After depleting ATP, the detection reagent was added to the reaction to detect luminescing ADP (Supplementary Figure S1, available online). Upon PIM blocking, either by inhibitor (HuscFvs or small chemical ADZ1208), PIM kinase is unable to utilized ATP and hence ADP is not generated. The level of ATP remained in the reaction was high, on the other hand, the level of generated ADP in the reaction was low. After the ATP depletion, the remaining ADP was scarce; and thus, low luminescent signal is generated.

HuscFvs at 2, 4, and 8 µM were mixed with 6 ng active PIM2 (SignalChem, Richmond, BC, Canada) in 1× kinase buffer in 384-well-white plate (Corning) (triplicate wells). Control HuscFv (8 µM) and pan-PIM small inhibitor, AZD1208 (Sigma, Merck KGaA) (50 and 200 nM) were included to the experiment as negative and positive PIM2 inhibition controls, respectively. Pim2 in buffer alone served as blank (non-treated control). After 1 h incubation at 37 °C, 1 µg S6K substrate (SignalChem), along with 5 µM ATP (Promega, Madison, WI, USA) were added to the reactions. After keeping at 37 °C for 1 h, the remaining ATP in each reaction was depleted by adding 5 µL of ADP-Glo™ reagent (Promega) to each reaction mixture and kept at room temperature for 40 min. Then, 10 µL of kinase detection reagent (Promega) was added. The luminescence was recorded at 30 min by using Synergy H1 (BioTek, Winooski, VT, USA) with 1 s integration time and gain 135. Two independent experiments were performed.

### 4.12. Statistical Analysis

Luminescence signals from individual treatment was filled in Prism 9.2 (Graphpad). One-way analysis of variance (one-way ANOVA) was used to compare luminescent signals between treatment groups. The multiple comparison of means was also calculated using Tukey’s method. *p*-values less than 0.05 were considered statistically different. 

## 5. Conclusions

PIM2 and other PIM kinases are rational targets of pan anti-cancer therapeutics as they involve in tumorigenesis and tumor progression of many cancers. Several small chemical drugs targeting the kinases have been developed, but their off-target toxicity limits their clinical application. In this study, fully human single-chain antibodies to PIM2 were generated using phage display technology. Recombinant PIM2 was used as an antigenic bait to fish out the rPIM2-bound phages from the human scFv (HuscFv) display phage library, of which some phages in the library displayed HuscFvs to human own proteins. HuscFvs produced by three *E. coli* clones infected with the HuscFv displaying phages bound also to native PIM2 from cancer cells. The HuscFvs presumptively interacted with the PIM2 at the ATP binding pocket and kinase active loop, common to all PIMs. They inhibit kinase activity of PIM2 *in vitro*. The fully human HuscFvs should be developed into cell-penetrating format (by linking molecularly the HuscFvs with human cells penetrating peptide or entrapping the HuscFvs in suitable biocompatible nanoparticles) and tested further towards clinical application as novel and safe pan-anti-cancer therapeutics.

## Figures and Tables

**Table 1:** Percent amino acid homology of the PIM2-bound-HuscFv sequences from the *huscfv*-phagemid-transformed-HB2151 *E. coli* Clones 3, 7, 10, 15, 28, 34, 37, 40 and 42 with the closest human V region frameworks (FRs).

*E. coli* Clone No.	Ig Domain	Closest Human V Region	Identity (%)	Amino Acid Homology with Human FRs (%)		
				FR1	FR2	FR3
3	VH	M99649 IGHV3-7*01	96.53	92.00	100.00	92.11
	VL	Z00023 IGKV4-1801	97.64	100.00	100.00	100.00
7	VH	M99660 IGHV3-23*01	100.00	100.00	100.00	100.00
	VL	X01668 IGKV3-11*01	97.85	100.00	100.00	94.44
10	VH	J04096 IGHV6-1*01	100.00	100.00	100.00	100.00
	VL	Z00013 IGKV1-0*01	93.19	84.62	94.12	97.22
15	VH	M99641 IGHV-18*01	98.61	96.00	100.00	100.00
	VL	X59315 IGKV1-39*01	98.92	92.31	100.00	100.00
28	VH	X62112 IGHV4-4*07	99.30	100.00	100.00	128.57
	VL	X59315 IGKV1-39*01	96.77	92.31	100.00	97.22
34	VH	X92255 IGHV4-34*03	97.89	100.00	100.00	94.59
	VL	X12686 IGKV3-20*01	91.49	96.15	88.24	91.67
37	VH	AC245166 IGHV3-23*04	100.00	100.00	100.00	100.00
	VL	M23090 IGKV3-15*01	95.70	96.15	94.12	94.44
40	VH	M99663 IGHV3-30*03	97.92	80.00	100.00	100.00
	VL	X12686 IGKV3-20*01	99.29	100.00	100.00	100.00
42	VH	X92343 IGHV1-46*01	99.65	96.00	100.00	100.00
	VL	M23090 IGKV3-15*01	96.06	96.15	100.00	88.89

Ig, immunoglobulin; FR, immunoglobulin framework region; VH, variable domain of heavy chain; VL, variable domain of light chain. Asterisk followed by two numbers indicates the allele polymorphism.

**Table 2:** PIM2 residues and site(s) that interacted with the HuscFv7, HuscFv34 and HuscFv37.

PIM2		HuscFv7		Interactive Bond
Residue	Region	Residue	Domain	
Y214		T28	VH-CDR1	Hydrogen
H215		T28	VH-CDR1	Hydrogen
A216		T28	VH-CDR1	Hydrogen
A187		S54	VHCDR2	Hydrogen
R65		Y59	VH-CDR2	Hydrogen
I63		Y105	VH-CDR3	Pi-alkyl
P64		Y105	VH-CDR3	Pi-alkyl
K40	ATP pocket	S106	VH-CDR3	Hydrogen
D198	Active loop	Y107	VH-CDR3	Hydrogen
E239		K111	VH-CDR3	Salt bridge
R201		D112	VH-CDR3	Hydrogen
G199		Y113	VH-CDR3	Hydrogen
H212		D120	VH-CDR3	Hydrogen
R65		W241	VL-CDR3	Hydrogen, Pi-alkyl
		HuscFv34		
		Residue	Domain	
H63		F29	VH-FR1	Pi-alkyl
P64		F29	VH-FR1	Pi-alkyl
F43	ATP pocket	S30	VH-FR1	Hydrogen
R201		E50	VH-CDR2	Salt bridge, Attractive charge
D198	Active loop	N52	VH-CDR2	Hydrogen
F43	ATP pocket	H53	VH-CDR2	Hydrogen
S185		H53	VH-CDR2	Hydrogen
R201		S56	VH-CDR2	Hydrogen
P64		N76	VH-CDR4	Hydrogen
H212		R103	VH-CDR3	Pi-cation
R211		S163	VL-CDR1	Hydrogen
H212		S163	VL-CDR1	Hydrogen
R201		Y229	VL-CDR3	Hydrogen
H212		Y229	VL-CDR3	Hydrogen
		HuscFv37		
		Residue	Domain	
L37		N101	VH-CDR3	Hydrogen
D124		Y102	VH-CDR3	Hydrogen
A122, Q123		Y102	H-CDR3	Amide-pi
E131		F104	H-CDR3	Pi-anion
K40	ATP pocket	Y111	VH-CDR3	Hydrogen
E131		R170	VL-CDR1	Hydrogen
K132		R170	VL-CDR1	Hydrogen
G234		N171	VL-CDR1	Hydrogen
T130		N172	VL-CDR1	Hydrogen
K40	ATP pocket	Y189	VH-FR2	Hydrogen
K40	ATP pocket	T196	VL-CDR2	Hydrogen
D235		R206	VL-CDR4	Hydrogen
E239		R206	VL-CDR4	Salt bridge
S207		S207	VL-CDR4	Hydrogen

## Data Availability

All datasets presented in this study are included in the article.

## Supplementary Materials

The following is available online, Supplementary Figure S1: Principles of PIM kinase and PIM kinase inhibition assays.
