# Supplementary material for: Engineered Fully Human Single-Chain Monoclonal Antibodies to PIM2 Kinase
Source: Molecules. 2021 Oct 25;26(21):6436. doi: 10.3390/molecules26216436 (PMC8588357; doi:10.3390/molecules26216436)
Supplement: Supplementary file 1 [file molecules-26-06436-s001.zip › molecules-1438318-supplementary.pdf]

Supporting information for:

Engineered Fully Human Single-chain Monoclonal Antibodies to PIM2 Kinase

Kanasap Kaewchim [1, 2], Kittirat Glab-ampai [2], Kodchakorn Mahasongkram [2], Monrat Chulanetra [2], Watee Seesuay[2], Wanpen Chaicumpa [2] and Nitat Sookrung [2, 3,*]

[1] Graduate Program in Immunology, Department of Immunology, Faculty of Medicine Siriraj Hospital, Mahidol University, Bangkok 10700, Thailand
[2] Center of Research Excellence on Therapeutic Proteins and Antibody Engineering, Department of Parasitology, Faculty of Medicine Siriraj Hospital, Mahidol University, Bangkok 10700, Thailand
[3] Biomedical Research Incubator Unit, Department of Research, Faculty of Medicine Siriraj Hospital, Mahidol University, Bangkok 10700, Thailand
* Correspondence: nitat.soo@mahidol.ac.th

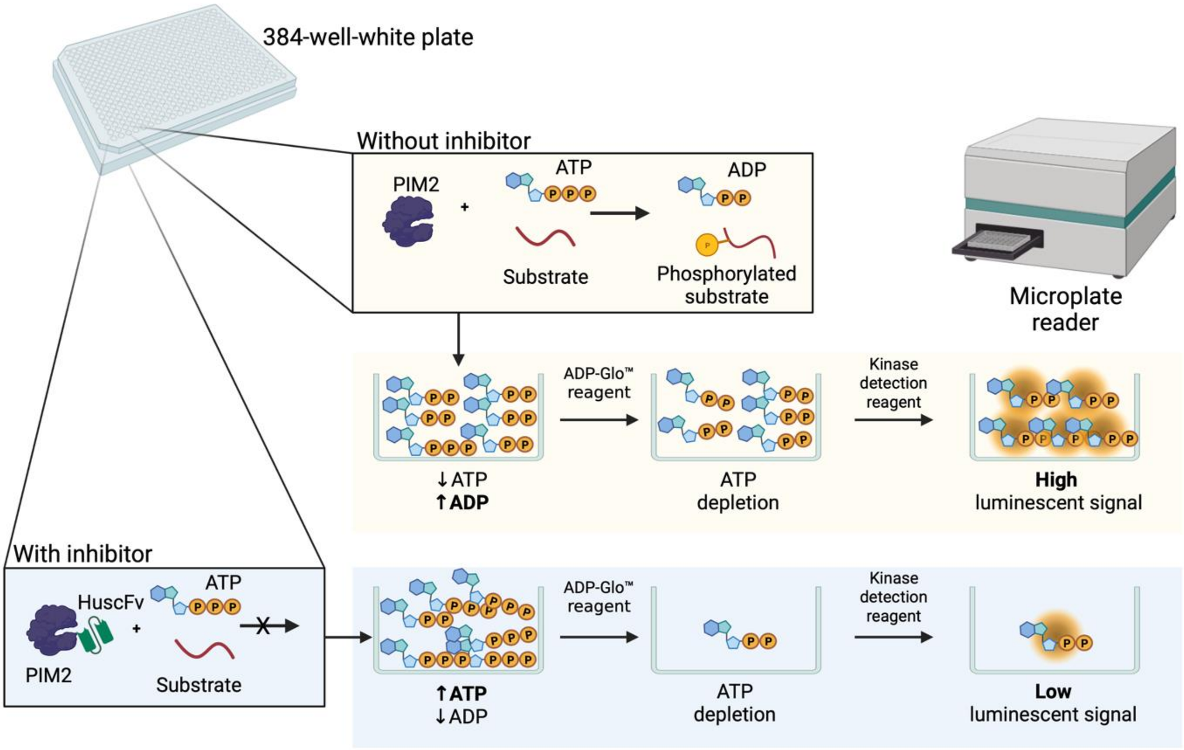

Supplementary Figure 1. Principles of PIM2 kinase and PIM2 kinase inhibition assays**.** PIM2 kinase phosphorylates the substrate using ATP resulting in ADP generation. After ATP depletion by ADP-Glo reagent, kinase detection reagent is added, and the luminescence signal is then detected. Upon blocking PIM2 kinase activity, either by HuscFvs or small chemical inhibitor, the kinase is unable to utilize ATP; hence, ADP is not generated. The level of ATP left in the reaction is high; on the other hand, the level of ADP generated in the reaction is low. After the ATP depletion by the ADP-Glo, the ADP in the reaction is scarce or negligible; and thus low luminescent signal is generated. The figure was created with BioRender.com.
